# Infant feeding knowledge and practice vary by maternal HIV status: a nested cohort study in rural South Africa

**DOI:** 10.1186/s13006-020-00317-5

**Published:** 2020-09-01

**Authors:** H. Manisha Yapa, Róisín Drayne, Nigel Klein, Jan-Walter De Neve, Kathy Petoumenos, Awachana Jiamsakul, Carina Herbst, Deenan Pillay, Frank A. Post, Till Bärnighausen

**Affiliations:** 1 The Kirby Institute, University of New South Wales Sydney, Sydney, Australia; 2 Africa Health Research Institute (AHRI), KwaZulu-Natal, South Africa; 3 School of Population Health & Environmental Sciences, King’s College London, London, UK; 4 UCL Great Ormond Street Institute of Child Health, London, UK; 5 Heidelberg Institute of Global Health (HIGH), Medical Faculty and University Hospital, University of Heidelberg, Heidelberg, Germany; 6 Division of Infection & Immunity, University College London, London, UK; 7 King’s College Hospital NHS Foundation Trust, London, UK; 8 Department of Global Health and Population, Harvard T.H. Chan School of Public Health, Boston, USA; 9 Institute for Global Health, University College London, London, UK

**Keywords:** Exclusive breastfeeding, HIV/AIDS, Clinical guidelines, Healthcare quality, Resource poor, Primary care

## Abstract

**Background:**

We investigate whether correct infant feeding knowledge and practice differ by maternal HIV status in an era of evolving clinical guidelines in rural South Africa.

**Methods:**

This cohort study was nested within the MONARCH stepped-wedge cluster-randomised controlled trial (www.clinicaltrials.gov: NCT02626351) which tested the impact of continuous quality improvement on antenatal care quality at seven primary care clinics in KwaZulu-Natal, from July 2015 to January 2017. Women aged ≥18 years at delivery were followed up to 6 weeks postpartum. Clinical data were sourced from *routine medical records* at delivery. *Structured interviews* at early postnatal visits and the 6-week postnatal immunisation visit provided data on infant feeding knowledge and feeding practices respectively. We measured the relationship between maternal HIV status and (i) correct infant feeding knowledge at the early postnatal visit; and (ii) infant feeding practice at 6 weeks, using Poisson and multinomial regression models, respectively.

**Results:**

We analysed data from 1693 women with early postnatal and 471 with 6-week postnatal interviews. HIV prevalence was 47% (95% confidence interval [CI] 42, 52%). Women living with HIV were more knowledgeable than women not living with HIV on correct infant feeding recommendations (adjusted risk ratio, aRR, 1.08, *p* <  0.001). More women living with HIV (33%; 95% CI 26, 41%) were not breastfeeding than women not living with HIV (15%; 95% CI 11, 21%). However, among women who were currently breastfeeding their infants, fewer women living with HIV (5%; 95% CI 2, 9%) mixed fed their babies than women not living with HIV (21%; 95% CI 14, 32%). In adjusted analyses, women living with HIV were more likely to avoid breastfeeding (adjusted relative risk ratio, aRRR, 2.78, *p* <  0.001) and less likely to mixed feed (aRRR 0.22, *p* <  0.001) than women not living with HIV.

**Conclusions:**

Many mothers in rural South Africa still do not practice exclusive breastfeeding. Women living with HIV were more knowledgeable but had lower overall uptake of breastfeeding, compared with women not living with HIV. Women living with HIV were also more likely to practice exclusive breastfeeding over mixed feeding if currently breastfeeding. Improved approaches are needed to increase awareness of correct infant feeding and exclusive breastfeeding uptake.

## Background

Exclusively breastfeeding infants for the first 6 months of life can be life-saving, have long-term health benefits [1–4], and is aligned with sustainable development goals (SDGs) [5]. Yet a potential barrier to uptake of breastfeeding in HIV-endemic settings is risk of mother-to-child transmission of HIV (MTCT) which is correlated with maternal viral load [6, 7]. Effective antiretroviral therapy (ART) during pregnancy and breastfeeding minimises MTCT [7]. Exclusive breastfeeding also lowers MTCT risk compared with mixed feeding (breastmilk with other foods or fluids) even with untreated maternal HIV [4, 8]. An important caveat is HIV reservoirs in latent and active CD4+ T cells in breastmilk even among women on suppressive ART [9]. The benefits of exclusive breastfeeding in resource-poor settings outweigh any risks (including concerns of micronutrient deficiency without supplementary feeds after 4 months of age) [10], supporting recommendations of exclusive breastfeeding for all infants until 6 months of age regardless of maternal HIV status [11, 12].

Although the rapid evolution of clinical guidelines may challenge their real-time implementation, infant feeding guidelines and elimination of mother-to-child transmission of HIV (eMTCT) guidelines must be applied concurrently for maximal impact, particularly in HIV-endemic settings. South Africa, the highest HIV burden country in the world, has further changed infant feeding and ART guidelines since 2015 alongside major efforts to improve exclusive breastfeeding (6 months for all women) since 2011 [13–15]. These changes included revising the total breastfeeding duration to 24 months for all women regardless of HIV status aligned with WHO guidelines [16], CD4 eligibility expansions for ART — Option B+ for pregnant and breastfeeding women from January 2015 [17], and Universal Test and Treat for all people living with HIV from September 2016 [18] — and more frequent HIV viral load monitoring [17, 18].

Within the context of these new guidelines we anticipate more postpartum women to initiate and sustain exclusive breastfeeding in South Africa, because they may be more confident in suppressive ART; be aware of how maternal viral load influences HIV transmission, and be aware of the dangers of mixed feeding and benefits of exclusive breastfeeding. However, despite substantial improvements in exclusive breastfeeding initiation [19–22], early cessation of exclusive breastfeeding remains a problem [22–25].

The antenatal period is a crucial phase during which women engaging with healthcare services may become aware of their HIV status and receive critical information on HIV care and infant feeding. Women’s knowledge of infant feeding recommendations, knowledge of HIV, and actual feeding practices therefore reflect quality of healthcare services. We tested whether a continuous quality improvement (CQI) intervention (MONARCH, www.clinicaltrials.gov: NCT02626351) could improve antenatal HIV services in public sector primary care clinics [26, 27]. The pre-registered primary endpoints were HIV viral load monitoring among pregnant women living with HIV and repeat HIV testing among pregnant women not living with HIV; the primary findings are reported elsewhere: briefly, CQI improved viral load monitoring but not repeat HIV testing [27].

The aims of this paper are to examine, among women recruited to the MONARCH trial (1) whether knowledge of infant feeding recommendations differs by maternal HIV status; and (2) whether infant feeding practice differs by maternal HIV status.

## Methods

### Study design

The CQI intervention targeted health workers providing antenatal services at seven participating primary care clinics in northern KwaZulu-Natal, located within and adjoining the Africa Health Research Institute (AHRI) population intervention platform surveillance area (PIPSA). The first six of these seven primary care clinics are all of the clinics located within the AHRI PIPSA geographic bounds, which formed the contiguous geographically designed study community for this study. The seventh clinic, located in the market town of Mtubatuba, was located outside the AHRI PIPSA geographic bounds. We included this clinic in our study, because it is the one primary clinic that people living in the AHRI PIPSA community frequently attend [26]. Details of the MONARCH stepped-wedge cluster-randomised controlled trial are reported elsewhere [26].

The present cohort study was nested within the parent trial. Eligible women were followed from delivery up to 6 weeks postpartum, between July 2015 and January 2017. Thus, the same version of guidelines on duration of exclusive breastfeeding (6 months for all women) and ART eligibility for pregnant and breastfeeding women (removal of CD4 count criteria, Option B+) applied to all women enrolled in this study.

### Participants

Women were aged ≥18 years at delivery and recruited at three time points independent of previous or future recruitment: delivery, the 3–6 day postnatal visit, and the 6-week postnatal immunisation visit [26]. Women who were recruited at more than one time point were linked within the study database. Women were recruited at delivery if they were resident within the AHRI population surveillance area during pregnancy or attended one of the seven study clinics during pregnancy [26]. At postnatal visits, women who attended a study clinic were recruited regardless of their antenatal clinic or area of residency.

### Exposure

The main exposure of interest was maternal HIV status at delivery, as documented in the antenatal medical record.

### Endpoints

We considered the following two endpoints: (1) correct knowledge of infant feeding recommendations at an early postnatal visit (delivery or 3–6 days postpartum); and (2) self-reported uptake of feeding modalities in relation to exclusive breastfeeding at 6 weeks postpartum (see Table 1 for definitions). We also describe knowledge of HIV treatment and transmission as an exploratory analysis, by maternal HIV status.

**Table 1:** Study endpoint definitions

Outcome type	Definition
***Knowledge of infant feeding recommendations (early postnatal interviews)***	
This was a total score out of 3, each question coded as correct or incorrect, with a higher score indicating better knowledge:	
• defining exclusive breastfeeding correctly	
• identifying exclusive breastfeeding as the recommended feeding method for all infants	
• identifying exclusive breastfeeding as the recommended feeding method for HIV-exposed infants	
***Infant feeding practices (6-week postnatal interviews)***	
This was classified in three unordered categories:	
*(i) Exclusive breastfeeding defined as:*	
• currently breastfeeding; *and*	
• never administered other food or fluids to the infant	
*(ii) Mixed feeding defined as*:	
• currently breastfeeding; *and*	
• ever administered other food or fluids to the infant	
*(iii) Not currently breastfeeding:*	
• this included women who may have initiated breastfeeding and ceased prior to the 6-week interview as well as those who had exclusively replacement fed their infant since delivery	
***Knowledge of HIV treatment and transmission (6-week postnatal interviews)***	
This was a total score out of 8 questions, each coded as correct or incorrect, with a higher score indicating better knowledge:	
• HIV viral load knowledge (meaning of a suppressed viral load)	
• role of a suppressed viral load in sexual transmission	
• role of a suppressed viral load in MTCT through breastmilk	
• when to test for HIV	
• the role of ART in improving health (2 questions)	
• that ART is lifelong	
• the role of CD4 count measurement	

*ART* antiretroviral therapy, *MTCT* mother-to-child transmission of HIV

### Data sources

Clinical data including HIV status were sourced from antenatal medical records. Structured interviews of consenting women conducted at delivery, the 3–6 day postnatal visit and the 6-week postnatal visit were sourced for demographic data and the endpoints listed above. The delivery and 3–6 day postnatal interviews were identical and included a theme on *knowledge of infant feeding* (Table S1), whereas the 6-week postnatal interview covered *knowledge of HIV treatment and transmission*, and *self-reported practices of infant feeding* (Table S1) [26]. We selected the 6-week interview for HIV treatment and transmission knowledge questions for the following reasons: (i) we were concerned women may find such questions too stressful to handle shortly after giving birth, and (ii) the 6-week postnatal visit was the next scheduled routine clinic visit (aligned with infant immunisation) following the 3–6 day postnatal visit. Given our recruitment method, some women were interviewed at delivery *and* the 3–6 day postnatal visit, whereas others were interviewed at only one of these early postnatal visits.

Participants were included in the analysis for endpoint 1 (feeding knowledge) if they had (i) a medical record available; *and* (ii) a delivery or 3–6 day (early postnatal) interview available. Where both delivery and 3–6 day interviews were available, the delivery interview was analysed as it was the earliest opportunity to measure maternal feeding knowledge. Participants were included in the analysis for endpoint 2 (feeding practice) if they had (i) a medical record available; *and* (ii) a delivery or 3–6 day interview available; *and* (iii) a 6-week postnatal interview available. Knowledge of HIV treatment and transmission was analysed only among women included for endpoint 2.

### Statistical analyses

We used Poisson regression to determine the association between HIV status and correct knowledge of infant feeding, because our knowledge outcome was a count. The regression model generated risk ratios (RR). We then used a multinomial regression model [28] to determine the association between HIV status and three unordered categories of infant feeding (exclusive breastfeeding, mixed feeding, not currently breastfeeding). Not currently breastfeeding and mixed feeding were each compared against the base category of exclusive breastfeeding. Coefficients were generated for the effect of each independent variable (including HIV status) on each feeding category relative to the base category of exclusive breastfeeding (relative risk ratios, RRR).

All ‘basic’ models included adjustments for maternal age and education status. Models for feeding practice also included infant feeding knowledge. Adjusted models were complete case analyses with covariates for parity, relationship status, employment status, household assets, CQI exposure, and calendar time. We used household assets as a proxy for household income given the large number of missing responses to the latter. We also separately explored knowledge of HIV treatment and transmission (from 6-week postnatal interviews) to support our interpretation of feeding practices. We clustered standard errors by first attended antenatal clinic (i.e., the seven study clinics and a category for “other” clinics). Based on our eligibility criteria for recruitment at delivery, not all women attended a study clinic at their first antenatal visit. The first antenatal clinic was selected as that was the first opportunity for influencing the reported outcomes.

Sensitivity analyses: in the adjusted models for infant feeding knowledge (early postnatal interviews) and infant feeding practices (6-week postnatal interviews) we substituted household income for household assets to examine the robustness of our main findings.

Statistical significance was defined at the *α* = 0.05 level. Stata version 15.0 (StataCorp. 2017. *Stata Statistical Software: Release 15*) was used for all analyses.

## Results

Of 3147 participants in the parent trial, 2498 had a medical record available. Of these, 1693 women (68%) completed a delivery and/or 3–6 day (early postnatal) interview; 516/1693 completed both delivery and 3–6 day interviews; 471/1693 women (28%) completed a 6-week postnatal interview. Participant characteristics at the early postnatal visit of those with and without a 6-week postnatal interview were similar (Table S2). Most women (86%) attended a study clinic for their first antenatal care (ANC) visit.

Median age was 25 years (interquartile range [IQR], 21–30). Median gestation at first ANC visit was 19 weeks (IQR 15–24 weeks). Most women were unemployed and were not living with their partner (Table 2). Women living with HIV were less educated, had less household wealth and more children than women not living with HIV; however, more women living with HIV were employed than women not living with HIV (Table 2). HIV prevalence at delivery was 47% (95% CI 42, 52%). Of women living with HIV, 93% had at least one documented ART prescription during pregnancy and 56% had at least one viral load measured during pregnancy. Of viral loads performed 53% had a documented result, 83% of which were suppressed < 200 copies/mL.

**Table 2:** Participant characteristics overall and by HIV status among 1693^c^ women with early postnatal interviews

Characteristic	Overall	Women not living with HIV	Women living with HIV	***p***-value^*^
**Number**	**1680**	**895**	**785**	
Age, years (IQR)	25 (21–30)	23 (20–27)	28 (23–32)	< 0.001
Education, *n* (%)^a^				0.003
Less than high school	730 (43.6)	355 (39.9)	375 (47.8)	
High school or above	945 (56.1)	539 (60.0)	406 (51.7)	
Missing	5 (0.3)	1 (0.1)	4 (0.5)	
Employment, *n* (%)^a^				0.001
Employed/ other	221 (13.1)	88 (9.8)	133 (16.9)	
Unemployed	1453 (86.5)	803 (89.8)	650 (82.9)	
Missing	6 (0.4)	4 (0.4)	2 (0.3)	
Household assets^‡^, *n* (%)^a^				0.003
≥ 15 assets	551 (32.9)	321 (36.1)	230 (29.3)	
< 15 assets	1129 (67.1)	574 (63.9)	555 (70.7)	
Household income, *n* (%)				< 0.001
Family income ≥ R2000	714 (42.5)	380 (42.5)	334 (42.5)	
Family income < R2000	620 (36.9)	292 (32.5)	328 (41.8)	
Missing	346 (20.6)	223 (25.0)	123 (15.7)	
Relationship status, *n* (%)^a^				0.559
Married/ living with partner/ other	278 (16.6)	136 (15.2)	142 (18.2)	
Not married and not living with partner	1397 (83.2)	757 (84.6)	640 (81.6)	
Missing	5 (0.2)	2 (0.2)	3 (0.3)	
Number of children, *n* (%)^a^				< 0.001
More than 1 child	1061 (63.1)	444 (49.5)	617 (78.6)	
One child	615 (36.6)	449 (50.3)	166 (21.1)	
Missing	4 (0.2)	2 (0.2)	2 (0.3)	
Drinking water source^b^, *n* (%)^a^				0.325
Piped water in or on property	617 (36.7)	327 (36.7)	290 (36.8)	
Communal water pipe, bore hole, tank	883 (52.5)	463 (51.6)	420 (53.6)	
Other (stream/ dam/ purchase)	180 (10.7)	105 (11.7)	75 (9.6)	
Time travelled to clinic during pregnancy^b^, *n* (%)^a^				0.006
< 15 min	209 (12.4)	127 (14.1)	82 (10.5)	
15–30 min	750 (44.6)	388 (43.4)	362 (46.0)	
31–60 min	479 (28.5)	256 (28.6)	223 (28.5)	
> 60 min	222 (13.2)	111 (12.5)	111 (14.1)	
Missing	20 (1.2)	13 (1.5)	7 (0.9)	
Exposure to CQI during pregnancy, *n* (%)^a^				0.002
Unexposed	975 (58.3)	536 (60.3)	439 (56.1)	
Exposed	705 (41.7)	359 (39.7)	346 (43.9)	

*CQI* continuous quality improvement, *IQR* interquartile range

^*^Pearson’s Chi square test for difference between women living with HIV vs women not living with HIV

^a^All proportions are adjusted for clustering by first attended antenatal clinic

^b^Drinking water source and time travelled to clinic were excluded from final adjusted regression models by a backwards stepwise regression process to achieve model parsimony. A significance level of 0.05 was used during the process of comparing model fit

^c^HIV status missing in *n* = 13 early postnatal interviews

^‡^ Household assets were a checklist of household items (types of furniture, appliances, livestock etc) as indicators of household wealth in the AHRI population surveillance area. For the present analysis, ‘wealthy’ households were classified as those having at least 15 assets and poor households were those with < 15 assets. Household assets were included in adjusted models as a proxy for household income given the degree of missingness in the income variable

### HIV status and infant feeding knowledge (early postnatal interviews)

Most women living with HIV and women not living with HIV responded correctly to infant feeding knowledge questions (Table 3). Women living with HIV were more likely to be knowledgeable on infant feeding recommendations in basic (RR 1.09; 95% CI 1.07, 1.11) and adjusted (aRR 1.08; 95% CI 1.06, 1.09) regression models which included a covariate for parity (Table S3). This may be due to differences in awareness of the definition of exclusive breastfeeding and feeding recommendations for HIV-exposed infants (Table 3).

**Table 3:** Descriptive outcomes by HIV status: correct knowledge of infant feeding and infant feeding practices

**Correct feeding knowledge: early postnatal interview (*n =*** **1693)**^**a**^				
**Outcome**	**Overall** ***N*** **= 1680**	**Women not living with HIV** ***N*** **= 895**	**Women living with HIV** ***N*** **= 785**	***p*** - value^*^
	*n* (%)^**d**^	*n* (%)^**d**^	*n* (%)^**d**^	
Individual questions				
Definition of exclusive breastfeeding^b^	1617 (96.2%)	848 (94.7%)	769 (98.0%)	0.001
Infant feeding recommendations in general^c^	1612 (96.0%)	857 (95.7%)	755 (96.2%)	0.778
Infant feeding for women with HIV^b^	1391 (82.7%)	673 (75.0%)	718 (91.4%)	< 0.001
All responses missing	6 (0.4%)	4 (0.4%)	2 (0.3%)	
Total score				< 0.001
0–1 out of 3 correct	53 (2.3%)	36 (4.0%)	17 (2.2%)	
2 out of 3 correct	296 (17.7%)	222 (25.0%)	74 (9.5%)	
3 out of 3 correct	1331 (79.1%)	637 (71.0%)	694 (88.4%)	
**Feeding practice: 6-week postnatal interview (*n*** **= 471)**^**a**^				
	**Overall** ***n*** **= 467**	**Women not living with HIV** ***n*** **= 236**	**Women living with HIV** ***n*** **= 231**	***p*** - value^*^
	*n* (%)^**d**^	*n* (%)^**d**^	*n* (%)^**d**^	
				< 0.001
Exclusive breastfeeding	302 (64.5%)	156 (66.0%)	146 (63.0%)	
Mixed feeding	49 (10.5%)	42 (17.9%)	7 (3.0%)	
Not currently breastfeeding	112 (24.1%)	36 (15.3%)	76 (33.0%)	
Missing	4 (0.9%)	2 (0.9%)	2 (0.9%)	

^*^Pearson’s Chi square test for difference between women living with HIV vs women not living with HIV

^a^ HIV status missing in *n* = 13 early postnatal interviews and *n* = 4 6-week postnatal interviews

^b^Missing response in 8. Missing responses were coded as incorrect for the total score variable

^c^Missing response in 6. Missing responses were coded as incorrect for the total score variable

^d^Proportions are adjusted for clustering by first attended antenatal clinic

Not currently breastfeeding includes those who exclusively replacement fed their infants and those who ceased breastfeeding prior to the 6-week postnatal interview

Our findings remained robust to substituting household income for household assets in the adjusted model (aRR 1.08; 95% CI 1.06, 1.10), with a similar range of uncertainty (Table S3).

### HIV status and infant feeding practice (6-week postnatal interviews)

#### Uptake of any breastfeeding

Overall 351/467 (75%) women were breastfeeding (including exclusive breastfeeding and mixed feeding). Fewer women living with HIV (66%) were breastfeeding at 6 weeks compared with women not living with HIV (84%), Table 3.

#### Uptake of exclusive breastfeeding

**Fig. 1:**
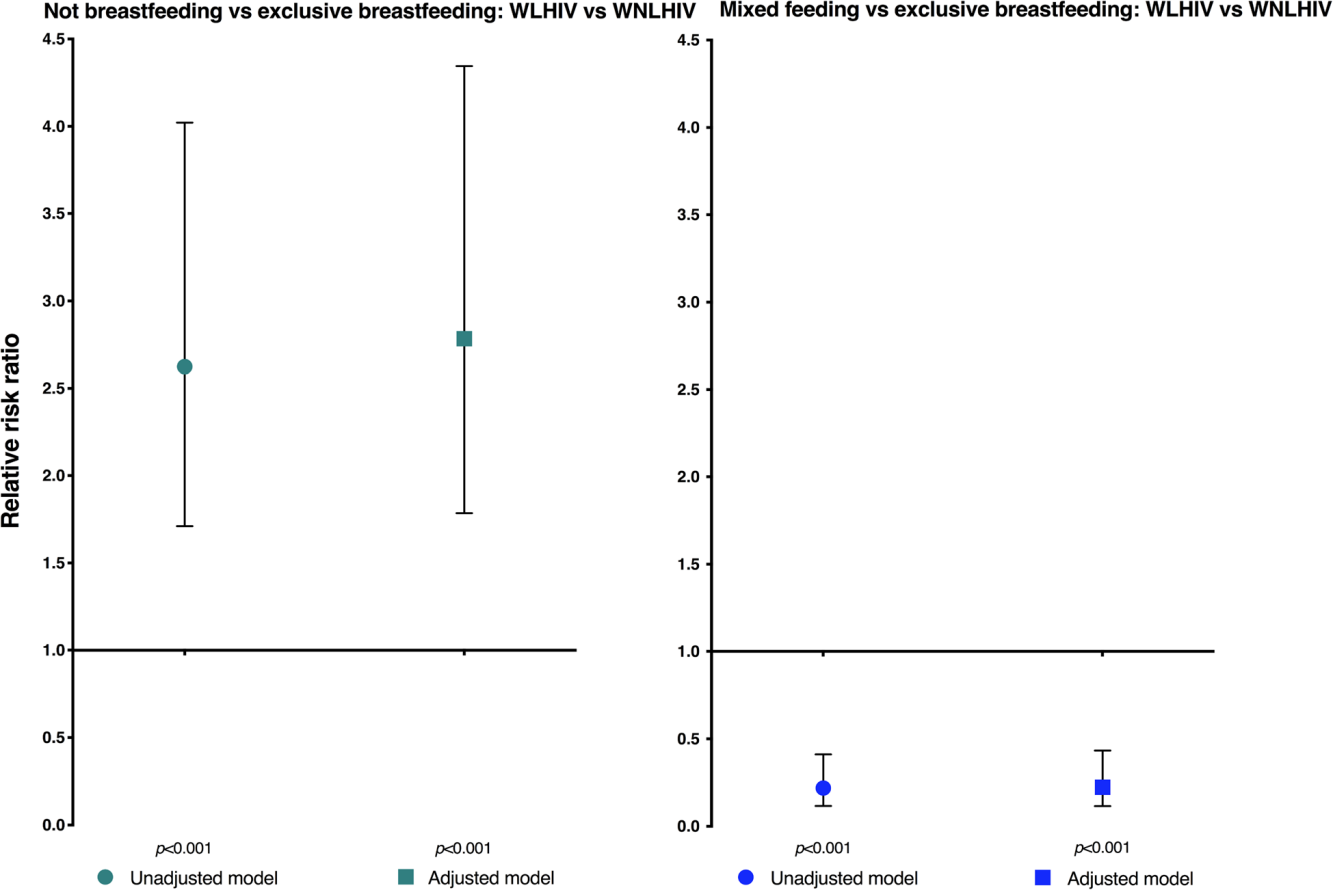
Association between maternal HIV status and infant feeding practices. Graphs depict outputs from unadjusted and adjusted multinomial regression models comparing (i) not breastfeeding vs exclusive breastfeeding, and (ii) mixed feeding vs exclusive breastfeeding. The unadjusted (basic) model contains a priori covariates for maternal age, education and knowledge. The adjusted model contains additional covariates for parity, employment, relationship status, household assets, CQI exposure, and time. Both unadjusted and adjusted models account for clinic-level clustering of outcomes. CQI, continuous quality improvement; WLHIV, women living with HIV; WNLHIV, women not living with HIV

Although similar proportions of *all* women living with HIV (63%) and women not living with HIV (66%) respectively practised exclusive breastfeeding, there were key differences by HIV status in mixed feeding and not currently breastfeeding (Table 3). In regression models relative to the baseline feeding category exclusive breastfeeding, women living with HIV (versus women not living with HIV) were more likely not to breastfeed in basic (RRR 2.62; 95% CI 1.71, 4.02) and adjusted (aRRR 2.78; 95% CI 1.78, 4.34) models. Moreover, women living with HIV were less likely to mixed feed their babies in basic (RRR 0.22; 95% CI 0.12, 0.41) and adjusted (aRRR 0.22; 95% CI 0.11, 0.43) models, see Fig. 1 and Table S3.

Our findings remained robust when we substituted household income for household assets in the adjusted model, albeit with a wider range of uncertainty: relative to the baseline feeding category exclusive breastfeeding, women living with HIV (versus women not living with HIV) were more likely not to breastfeed (aRRR 3.90; 95% CI 2.44, 6.24) and were less likely to mixed feed their babies (aRRR 0.26; 95% CI 0.11, 0.61), see Table S3.

#### Knowledge of HIV treatment and transmission (6-week postnatal interviews)

**Fig. 2:**
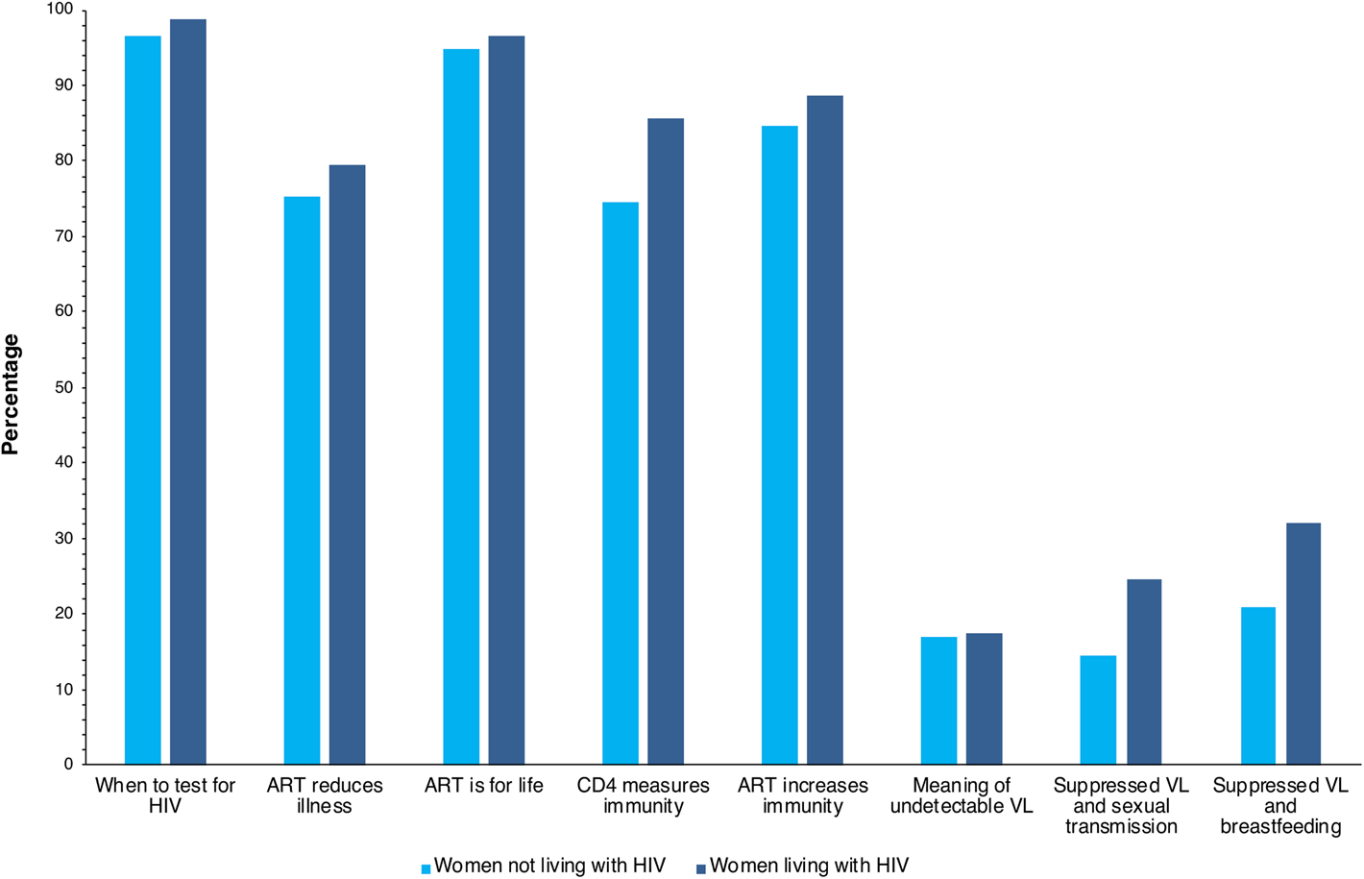
Proportion of women not living with HIV and women living with HIV with a correct response to each of eight questions on HIV treatment and transmission. ART, antiretroviral therapy; VL, HIV viral load; HIV status: at delivery

Most women living with HIV and women not living with HIV responded correctly to questions on HIV testing, the role of ART and the role of CD4 count. More women living with HIV responded correctly than did women not living with HIV to each question (Fig. 2). However, knowledge of viral load and its role in HIV transmission was poor, even among women living with HIV.

#### Other factors associated with feeding practice

Unemployed women were more likely to exclusively breastfeed than not breastfeed (aRRR for not breastfeeding vs exclusive breastfeeding 0.39; 95% CI 0.20, 0.76). Although infant feeding knowledge was associated with lower likelihood of mixed feeding vs exclusive breastfeeding in the basic model (RRR 0.50; 95% CI 0.30, 0.84), the effect size did not persist in the adjusted model and is of unclear significance. HIV treatment and transmission knowledge scores and other factors were not associated with infant feeding modality at commonly used benchmarks of statistical significance.

#### Reasons for infant feeding choice

Of 112 participants not currently breastfeeding, 43 provided reasons: lack of milk (26%), return to work (21%), and being too busy (12%); of the 28 women living with HIV, 21% cited HIV as a reason for not breastfeeding. Among 49 participants who were mixed feeding, reasons included lack of milk (47%), crying baby (33%), and return to work (4%).

## Discussion

Using data on 1693 women enrolled in our parent trial in rural South Africa, we demonstrate differences in knowledge of correct infant feeding recommendations, and self-reported infant feeding practices between mothers with and without HIV. Although women living with HIV were more knowledgeable on infant feeding recommendations, they were less likely to breastfeed than women not living with HIV. Conversely, among those currently breastfeeding, women living with HIV were more likely to exclusively breastfeed than mixed feed. Knowledge of the meaning and role of viral load suppression was poor, even among women living with HIV, and not associated with feeding practice. Our findings are important for health policy as future areas for targeted interventions.

Overall exclusive breastfeeding uptake in our study was similar to other studies from South Africa and elsewhere [19, 20, 23, 25, 29]. Our study mirrors two other studies on differences in exclusive breastfeeding uptake among women living with HIV compared with women not living with HIV in the Option B+ era [19, 20], despite being nested within a trial specifically targeting antenatal care quality for all women.

We postulate several reasons for these differences in infant feeding knowledge and practice. *First*, factors at health service level may have resulted in different delivery of feeding messages and support to women living with HIV compared with women not living with HIV. Importantly, there was no evidence of a CQI spillover effect from the main trial, and this is potentially attributable to insufficient time to improve infant feeding during the CQI intervention (due to time constraints of the study design and emphasis on primary endpoints). There may have been more opportunities for health workers to engage with women living with HIV (e.g. due to higher frequency of clinical consultations for HIV), or women not living with HIV may have paid less attention to messages due to less perceived relevance to themselves. Gaps in health worker understanding of the scientific rationale for exclusive breastfeeding among women living with HIV and women not living with HIV [30, 31], or inadequate breastfeeding support by health workers may have also contributed [32]. Consistent messaging and support to all women, regardless of their HIV status, are critical in an HIV hyperendemic setting such as this [33] where HIV seroconversion during the breastfeeding period is a real concern and risk of MTCT is high [34].

*Second*, there may be other individual- or community-level factors that transcend availability of correct information, although correct knowledge of infant feeding guidelines [19, 35–37] and quality of health worker messaging [19, 20, 23, 25, 38] influence individual feeding choice and duration. One factor is employment status [14, 20, 22, 39], especially as financial pressures may compel return to work shortly after birth without paid maternity leave [14, 40] and women may prefer replacement feeds over expressed breastmilk when returning to work or study [38]. Another is a limited support network for breastfeeding: most women in our study were not living with their partner, and poverty and unemployment may hinder marriage given traditional customs of bridewealth [41]. Among those who initiate breastfeeding, factors that may contribute to early cessation include cultural beliefs, stigma, HIV status disclosure, maternal mental health concerns, lack of a supportive workplace, and lack of paid maternity leave [14, 19, 21, 22, 29, 40]. Family pressures may also override women’s feeding choice [25, 30, 38, 39].

Finally, even among women living with HIV, awareness of the role of HIV viral load in treatment response and transmission was low. This knowledge gap — despite established South African national guidelines recommending HIV viral load monitoring for individuals on ART [42], and routinely available viral load monitoring at all facilities in the area – may be attributable to underutilisation of viral load monitoring as identified in our parent trial [27]. Whilst we expect better HIV treatment and transmission knowledge among women living with HIV, both groups of women were knowledgeable about HIV testing, the role of ART, and what a CD4 count means, indicating wide reach of general HIV treatment messages in this HIV hyperendemic community. Although HIV treatment and transmission knowledge were not associated with feeding practice, correct information on viral load is critical for ART adherence and reassurance on the safety of exclusive breastfeeding.

We add to the emerging evidence of infant feeding knowledge and practice in South African primary healthcare services after establishment of landmark HIV treatment guidelines [17, 18]. The limited maternal awareness of HIV viral load highlights the need for updating public health messages alongside rigorously implementing ART guidelines at health facilities. Methodological strengths of our study include maternal HIV status sourced directly from clinical records, detailed sociodemographic data from interviews, and a stringent definition of exclusive breastfeeding. By measuring feeding knowledge prior to feeding practice, we reduced knowledge recall bias.

There are some limitations to our study. *First*, our follow-up period was limited to 6 weeks and the total breastfeeding duration in our cohort is unknown. *Second*, social desirability biases may have influenced self-reported feeding practices. *Third*, the relatively small sample size of 6-week postnatal interviews may have reduced statistical power. *Fourth*, only a small subset of participants provided reasons for their feeding practices, and those results must be interpreted with caution.

Areas for further research include integrating maternal and child services, and interdisciplinary interventions to sustain exclusive breastfeeding. Longitudinal studies in the “treat all” era, on actual infant feeding practice up to 2 years postpartum concurrently with regular postpartum HIV testing for women not living with HIV, viral load monitoring for women living with HIV, and early diagnosis of HIV-exposed infants are needed.

## Conclusions

We found differences in infant feeding knowledge and practice among women living with HIV and women not living with HIV. We also found poor knowledge of the role of HIV viral load in monitoring treatment response and transmission. These findings may be due to differences in quality of health worker messages on feeding and low utilisation of HIV viral load in clinical practice.

A multifactorial approach is encouraged. These include enhanced health worker training and supervision on adherence to infant feeding and HIV treatment guidelines, education interventions targeting the wider community including family members, and work environments conducive to breastfeeding. We recommend routine programme evaluation indicators on infant feeding modality up to 2 years postpartum. Areas for future research include longitudinal studies on MTCT during the entire breastfeeding period.

## Supplementary information

**Additional file 1: Table S1.** Structured interview themes reviewed for knowledge and uptake outcomes. **Table S2.** Participant characteristics by availability of a 6-week postnatal interview. **Table S3.** Regression model outputs

## Data Availability

Fully anonymised data are available from the authors upon reasonable request. Access to datasets will be provided by the AHRI research data management team via the AHRI data repository at www.data.africacentre.ac.za

## Abbreviations

ART: Antiretroviral therapy
CQI: continuous quality improvement
DoH: South African national Department of Health
MTCT: Mother-to-child transmission of HIV
eMTCT: Elimination of mother-to-child transmission of HIV

## References

[1] Kramer MS, Kakuma R (2004). The optimal duration of exclusive breastfeeding: a systematic review. Adv Exp Med Biol.

[2] Bar S, Milanaik R, Adesman A (2016). Long-term neurodevelopmental benefits of breastfeeding. Curr Opin Pediatr.

[3] Victora CG, Vaughan JP, Lombardi C, Fuchs SMC, Gigante LP, Smith PG (1987). Evidence for protection by breast-feeding against infant deaths from infectious diseases in Brazil. Lancet.

[4] Coovadia HM, Rollins NC, Bland RM, Little K, Coutsoudis A, Bennish ML (2007). Mother-to-child transmission of HIV-1 infection during exclusive breastfeeding in the first 6 months of life: an intervention cohort study. Lancet.

[5] United Nations (2017). Sustainable development goals: breastfeeding is ‘smartest investment’ families, communities and countries can make – UN.

[6] Warszawski J, Tubiana R, Le Chenadec J, Blanche S, Teglas JP, Dollfus C (2008). Mother-to-child HIV transmission despite antiretroviral therapy in the ANRS French perinatal cohort. AIDS.

[7] WHO (2015). Mother-to-child transmission of HIV.

[8] Iliff PJ, Piwoz EG, Tavengwa NV, Zunguza CD, Marinda ET, Nathoo KJ (2005). Early exclusive breastfeeding reduces the risk of postnatal HIV-1 transmission and increases HIV-free survival. AIDS.

[9] Van De Perre P, Rubbo P-A, Viljoen J, Nagot N, Tylleskar T, Lepage P (2012). HIV-1 Reservoirs in Breast Milk and Challenges to Elimination of Breast-Feeding Transmission of HIV-1. Sci Transl Med.

[10] Kramer MS, Kakuma R (2012). Optimal duration of exclusive breastfeeding. Cochrane Database Syst Rev.

[11] Jones G, Steketee RW, Black RE, Bhutta ZA, Morris SS (2003). Bellagio child survival study group: how many child deaths can we prevent this year?. Lancet.

[12] Tchakoute CT, Sainani KL, Osawe S, Datong P, Kiravu A, Rosenthal KL, et al. Breastfeeding mitigates the effects of maternal HIV on infant infectious morbidity in the Option B+ era. AIDS. 2018;32:2383–91. 10.1097/QAD.0000000000001974 30134300

[13] National Department of Health South Africa (2011). The Tshwane declaration of support for breastfeeding in South Africa. South Afr J Clin Nutr.

[14] du Plessis L, Peer N, Honikman S, English R. Breastfeeding in South Africa: are we making progress? In: Padarath A, King J, Mackie E, Casciola J, editors. South African Health Review. Durban: Health Systems Trust; 2016. p. 109-24.

[15] National Department of Health South Africa (2013). Infant and young child feeding policy.

[16] WHO (2016). Updates on HIV and infant feeding.

[17] National Department of Health South Africa (2015). National Consolidated Guidelines for the Prevention of Mother-to-Child Transmission of HIV (PMTCT) and the Management of HIV in Children, Adolescents and Adults.

[18] National Department of Health South Africa (2016). Implementation of the Universal Test and Treat strategy for HIV positive patients and differentiated care for stable patients.

[19] Mnyani CN, Tait CL, Armstrong J, Blaauw D, Chersich MF, Buchmann EJ (2017). Infant feeding knowledge, perceptions and practices among women with and without HIV in Johannesburg, South Africa: a survey in healthcare facilities. Int Breastfeed J.

[20] Horwood C, Haskins L, Engebretsen IM, Phakathi S, Connolly C, Coutsoudis A (2018). Improved rates of exclusive breastfeeding at 14 weeks of age in KwaZulu Natal, South Africa: what are the challenges now?. BMC Public Health.

[21] Bork K, Cames C, Cournil A, Musyoka F, Ayassou K, Naidu K, et al. Infant feeding modes and determinants among HIV-1– infected African women in the Kesho Bora study. J Acquir Immune Defic Syndr. 2013;62:109–18. 10.1097/QAI.0b013e318277005e 23075919

[22] Somé EN, Engebretsen IMS, Nagot N, Meda N, Lombard C, Vallo R, et al. Breastfeeding patterns and its determinants among mothers living with Human Immuno-deficiency Virus −1 in four African countries participating in the ANRS 12174 trial. Int Breastfeed J. 2017;12:22. 10.1186/s13006-017-0112-2 PMC5414228 28469697

[23] Goga AE, Doherty T, Jackson DJ, Sanders D, Colvin M, Chopra M (2012). Infant feeding practices at routine PMTCT sites, South Africa: results of a prospective observational study amongst HIV exposed and unexposed infants - birth to 9 months. Int Breastfeed J.

[24] Patil CL, Turab A, Ambikapathi R, Nesamvuni C, Chandyo RK, Bose A (2015). Early interruption of exclusive breastfeeding: results from the eight-country MAL-ED study. J Health Popul Nutr.

[25] West NS, Schwartz SR, Yende N, Schwartz SJ, Parmley L, Gadarowski MB, et al. Infant feeding by South African mothers living with HIV: implications for future training of health care workers and the need for consistent counseling. Int Breastfeed J. 2019;14:11. 10.1186/s13006-019-0205-1 PMC6376722 30815026

[26] Chetty T, Yapa HMN, Herbst C, Geldsetzer P, Naidu KK, De Neve J-W (2018). The MONARCH intervention to enhance the quality of antenatal and postnatal primary health services in rural South Africa: protocol for a stepped-wedge cluster-randomised controlled trial. BMC Health Serv Res.

[27] Yapa HMN, De Neve J-W, Chetty T, Herbst C, Post F, Cooper DA, et al. Does QI improve PMTCT processes in rural South Africa? A stepped-wedge cluster RCT. Conference on Retroviruses and Opportunistic Infections. Boston; 2018: Abstract #819.

[28] Hoffman SD, Duncan GJ (1988). Multinomial and conditional logit discrete-choice models in demography. Demography.

[29] Onono MA, Cohen CR, Jerop M, Bukusi EA, J.M. T: HIV serostatus and disclosure: implications for infant feeding practice in rural South Nyanza, Kenya. BMC Public Health 2014; 14:390. 10.1186/1471-2458-14-390 PMC4041135 24754975

[30] Jama NA, Wilford A, Masango Z, Haskins L, Coutsoudis A, Spies L (2017). Enablers and barriers to success among mothers planning to exclusively breastfeed for six months: a qualitative prospective cohort study in KwaZulu-Natal, South Africa. Int Breastfeed J.

[31] Nieuwoudt S, Manderson L (2018). Frontline health workers and exclusive breastfeeding guidelines in an HIV endemic south African community: a qualitative exploration of policy translation. Int Breastfeed J.

[32] Doherty T, Horwood C, Haskins L, Magasana V, Goga A, Feucht U (2020). Breastfeeding advice for reality: women’s perspectives on infant feeding support received in primary health care settings in South Africa. Matern Child Nutr.

[33] Vandormael A, Newell M-L, Bärnighausen T, Tanser F (2014). Use of antiretroviral therapy in households and risk of HIV acquisition in rural KwaZulu-Natal, South Africa, 2004–12: a prospective cohort study. Lancet Glob Health.

[34] Dinh TH, Delaney KP, Goga A, Jackson D, Lombard C, Woldesenbet S (2015). Impact of maternal HIV seroconversion during pregnancy on early mother to child transmission of HIV (MTCT) measured at 4-8 weeks postpartum in South Africa 2011-2012: a national population-based evaluation. PLoS One.

[35] Ndubuka J, Ndubuka N, Li Y, Marshall CM, Ehiri J (2013). Knowledge, attitudes and practices regarding infant feeding among HIV-infected pregnant women in Gaborone, Botswana: a cross-sectional survey. BMJ Open.

[36] Gewa CA, Chepkemboi J (2016). Maternal knowledge, outcome expectancies and normative beliefs as determinants of cessation of exclusive breastfeeding: a cross-sectional study in rural Kenya. BMC Public Health.

[37] Cresswell JA, Ganaba R, Sarrassat S, Cousens S, Some H, Diallo AH, et al. Predictors of exclusive breastfeeding and consumption of soft, semi-solid or solid food among infants in Boucle du Mouhoun, Burkina Faso: A cross-sectional survey. PLoS One. 2017;12:e0179593. 10.1371/journal.pone.0179593 PMC5480894 28640900

[38] Adeniyi OV, Ajayi AI, Issah M, Owolabi EO, Goon DT, Avramovic G, et al. Beyond health care providers’ recommendations: understanding influences on infant feeding choices of women with HIV in the Eastern Cape, South Africa. Int Breastfeed J. 2019;14:7. 10.1186/s13006-019-0201-5 PMC6357465 30733819

[39] Mensah KA, Acheampong E, Anokye FO, Okyere P, Appiah-Brempong E, Adjei RO (2017). Factors influencing the practice of exclusive breastfeeding among nursing mothers in a peri-urban district of Ghana. BMC Res Notes.

[40] Department of Labour SA (2014). Basic Guide to Maternity Leave.

[41] Rudwick S, Posel D. Contemporary functions of ilobolo (bridewealth) in urban South African Zulu society. J Contemp Afr Stud. 2014;32:118–36.

[42] National Department of Health South Africa (2010). The South African Antiretroviral Treatment Guidelines.

