# Supplementary material for: Infant feeding knowledge and practice vary by maternal HIV status: a nested cohort study in rural South Africa
Source: Int Breastfeed J. 2020 Sep 1;15:77. doi: 10.1186/s13006-020-00317-5 (PMC7466779; doi:10.1186/s13006-020-00317-5)
Supplement: Supplementary file 1 — Additional file 1: Table S1. Structured interview themes reviewed for knowledge and uptake outcomes. Table S2. Participant characteristics by availability of a 6-week postnatal interview. Table S3. Regression model outputs [file 13006_2020_317_MOESM1_ESM.docx]

## **Additional File 1**

**“Infant feeding knowledge and practice vary by maternal HIV status: a nested cohort study in rural South Africa”**

**Table S1. Structured interview themes reviewed for knowledge and uptake outcomes**

| **Interview type** | **Theme^*^** | **Questions** | **Responses** | |
| --- | --- | --- | --- | --- |
| Early postnatal interview: delivery or 3-6 day postnatal visit | | | | |
|  | Knowledge of infant feeding recommendations | | | |
|  | 1 | Based on information you have received, could you tell me what feeding method is recommended for newborns for the first 6 months of life? | | (select one)  Feeding of formula milk/ Exclusive breastfeeding/ Feeding of soft foods (e.g porridge, mashed potato)/ A mix of breastfeeding, formula milk and soft foods/ Don’t know |
|  | 2 | What does exclusive breastfeeding mean? | | (select one)  Infant receives breastmilk alone/ Infant receives mostly breastmilk with other feeds/ Infant receives no breastmilk/ Other (specify)/ Don’t know/ Refused |
|  | 3 | If the mother is HIV-infected, what is the recommended method to feed her baby during the first 6 months of life? | | (select one)  Exclusive breastfeeding/ Exclusive feeding of formula/ A mix of foods including breastmilk, formula, soft porridge/ Cow’s milk/ Other (specify)/ Don’t know/ Refused |
| 6-week postnatal interview | | | | |
|  | Infant feeding practices^**^ | | | |
|  | 1 | Are you breastfeeding your baby? | | (select one)  Yes/ No [if Yes, go to Q4] |
|  | 2 | (if “No” to Q1) Did you breastfeed your baby at any stage? | | (select one)  Yes/ No |
|  | 3 | What was the reason you made the choice to feed your baby? | | (select multiple)  I do not like breastfeeding/ I am too busy/ I have to go back to work/ I am sick/ I do not have enough milk/ My baby is crying all the time/ My family member advised me not to/ A healthcare worker advised me not to/ Due to my HIV status/ Other (specify)/ Refused |
|  | 4 | For how long did you breastfeed your baby? | | (select one)  Less than 1 week/ 1-2 weeks/ 3-4 weeks/ Stopped just before 6 weeks/ Still breastfeeding/ Never breastfed/ Don’t know/ Refused |
|  | 5 | Has your baby been given any other foods or fluids other than breastmilk? | | (select one)  Yes/ No/ Don’t know/ Refused |
|  | 6 | (if “Yes” to Q5) When did you start other foods or fluids? | | (select one)  Less than 1 week old/ 1-2 weeks old/ 3-4 weeks old/ 5-6 weeks old/ Don’t know/ Refused |
|  | 7 | What made you decide to start giving your baby other food or fluids? | | (select multiple)  It is common practice in my community/ I thought it was the right time/ I do not like breastfeeding/ I am too busy/ I have to go back to work/ I am sick/ I do not have enough milk/ My baby is crying all the time/ My family member advised me to/ A healthcare worker advised me to/ Due to my HIV status/ Other (specify)/ Refused |
|  | HIV treatment knowledge | | | |
|  | 1 | People should test for HIV only when they feel sick | | (select one)  Agree/ Disagree/ Don’t know/ Refused |
|  | 2 | The risk of falling ill is reduced with ARV treatment | | (select one)  Agree/ Disagree/ Don’t know/ Refused |
|  | 3 | When someone who takes ARV treatment feels better they can stop taking the treatment | | (select one)  Agree/ Disagree/ Don’t know/ Refused |
|  | 4 | The CD4 count indicates how well the body can fight against diseases | | (select one)  Agree/ Disagree/ Don’t know/ Refused |
|  | 5 | The goal of ARV treatment is to increase the ability of the body to fight against diseases | | (select one)  Agree/ Disagree/ Don’t know/ Refused |
|  | 6 | Having an undetectable viral load means that the virus has stopped multiplying | | (select one)  Agree/ Disagree/ Don’t know/ Refused |
|  | 7 | When the viral load is very low there is almost no risk of transmitting the virus during sexual intercourse | | (select one)  Agree/ Disagree/ Don’t know/ Refused |
|  | 8 | When the viral load is very low there is almost no risk of transmitting the virus during breastfeeding | | (select one)  Agree/ Disagree/ Don’t know/ Refused |

^*^Only themes used for study endpoints are detailed here. Questionnaires were administered in isiZulu. Blank full structured questionnaires used at the delivery, 3-6 day postnatal and 6- week postnatal visits are available on request

^**^Women were considered to be currently breastfeeding if they answered “Yes” to Q1. Women were considered to be exclusively breastfeeding their baby if they answered “Yes” to Q1, and “No” to Q5. Women were considered to be mixed feeding their baby if they answered “Yes” to Q1 and “Yes” to Q5. Women were not currently breastfeeding if they answered “No” to Q1, and included women who may have breastfed initially and stopped by the six-week interview, as well as those who had exclusively replacement fed their baby since birth.

**Table S2. Participant characteristics by availability of a 6-week postnatal interview.**

|  | | **6-week interview available (*n*=471)** | | | **6-week interview not available (*n*=1222)** | | |
| --- | --- | --- | --- | --- | --- | --- | --- |
| **Characteristic** | | **Women not living with HIV** | **Women living with HIV** | ***p*-value^*^** | **Women not living with HIV** | **Women living with HIV** | ***p*-value^*^** |
|  | | ***n*=236**^‡^ | ***n*=231**^‡^ |  | ***n*=659^‡‡^** | ***n*=554^‡‡^** |  |
| Age, years (IQR) ^‡^ | | 23 (20-28) | 28 (23-31) | <0.001 | 22 (20-27) | 28 (24-32) | <0.001 |
| Education, n (%)^#^ | |  |  | 0.157 |  |  | 0.020 |
|  | Less than high school | 84 (35.7%) | 109 (47.4%) |  | 271 (41.4%) | 266 (48.0%) |  |
|  | High school or above | 151 (63.8%) | 121 (52.2%) |  | 388 (58.6%) | 285 (51.4%) |  |
|  | Missing | 1 (0.4%) | 1 (0.4%) |  | 0 | 3 (0.5%) |  |
| Employment, n (%)^#^ | |  |  | 0.657 |  |  | <0.001 |
|  | Employed/ other | 31 (12.8%) | 37 (16.1%) |  | 57 (8.7%) | 96 (17.2%) |  |
|  | Unemployed | 204 (86.8%) | 194 (83.9%) |  | 599 (90.8%) | 456 (82.4%) |  |
|  | Missing | 1 (0.4%) | 0 |  | 3 (0.5%) | 2 (0.4%) |  |
| Household assets^‡§^, n (%)^#^ | |  |  | 0.033 |  |  | 0.049 |
|  | ≥15 assets | 92(39.1%) | 68 (29.6%) |  | 229 (35.0%) | 162 (29.2%) |  |
|  | <15 assets | 144 (60.9%) | 163 (70.4%) |  | 430 (65.0%) | 392 (70.8%) |  |
| Household income, n (%)^#^ | |  |  | 0.047 |  |  | <0.001 |
|  | Family income ≥R2000 | 104 (44.3%) | 100 (43.5%) |  | 276 (41.9%) | 234 (42.0%) |  |
|  | Family income <R2000 | 77 (32.3%) | 95 (40.9%) |  | 215 (32.6%) | 233 (42.2%) |  |
|  | Missing | 55 (23.4%) | 36 (15.7%) |  | 168 (25.5%) | 87 (15.8%) |  |
| Relationship status, n (%)^#^ | |  |  | 0.657 |  |  | 0.443 |
|  | Married/ living with partner/ other | 38 (16.2%) | 39 (17.0%) |  | 98 (14.8%) | 103 (18.7%) |  |
|  | Not married and not living with partner | 198 (83.8%) | 192 (83.0%) |  | 559 (84.9%) | 448 (81.0%) |  |
|  | Missing | 0 | 0 |  | 2 (0.3%) | 3 (0.4%) |  |
| Number of children, n (%)^#^ | |  |  | <0.001 |  |  | <0.001 |
|  | More than 1 child | 125 (52.8%) | 179 (77.4%) |  | 319 (48.3%) | 438 (79.2%) |  |
|  | One child | 111 (47.2%) | 51 (22.2%) |  | 338 (51.4%) | 115 (20.7%) |  |
|  | Missing | 0 | 1 (0.4%) |  | 2 (0.3%) | 1 (0.2%) |  |
| Drinking water source^**^, n (%)^#^ | |  |  | 0.512 |  |  | 0.564 |
|  | Piped water in or on property | 71 (30.2%) | 81 (34.8%) |  | 256 (39.0%) | 209 (37.7%) |  |
|  | Communal water pipe, bore hole, tank | 137 (58.3%) | 131 (57.0%) |  | 326 (49.2%) | 289 (52.2%) |  |
|  | Other (stream/ dam/ purchase) | 28 (11.5%) | 19 (8.3%) |  | 77 (11.8%) | 56 (10.1%) |  |
| Time travelled to clinic during pregnancy^**^, n (%)^#^ | |  |  | 0.412 |  |  | 0.020 |
|  | <15 min | 27 (11.1%) | 14 (6.1%) |  | 100 (15.1%) | 68 (12.3%) |  |
|  | 15-30 min | 110 (46.8%) | 114 (49.1%) |  | 278 (42.2%) | 248 (44.7%) |  |
|  | 31-60 min | 62 (26.4%) | 69 (30.0%) |  | 194 (29.4%) | 154 (27.9%) |  |
|  | >60 min | 34 (14.5%) | 32 (13.9%) |  | 77 (11.8%) | 79 (14.1%) |  |
|  | Missing | 3 (1.3%) | 2 (0.9%) |  | 10 (1.5%) | 5 (0.9%) |  |
| Exposure to CQI during pregnancy^‡^, n (%)^#^ | |  |  | 0.178 |  |  | 0.009 |
|  | Unexposed | 153 (65.1%) | 139 (60.4%) |  | 383 (58.6%) | 300 (54.3%) |  |
|  | Exposed | 83 (34.9%) | 92 (39.6%) |  | 276 (41.4%) | 254 (45.7%) |  |

CQI, continuous quality improvement; IQR, interquartile range

^#^ All proportions are adjusted for clustering by first attended antenatal clinic

^*^ Pearson’s Chi square test for difference between women living with HIV vs women not living with HIV

^**^ Drinking water source and time travelled to clinic were excluded from final adjusted regression models by a backwards stepwise regression process to achieve model parsimony. A significance level of 0.05 was used to compare model fit

^§^ Household assets were a checklist of household items (types of furniture, appliances, livestock etc) as indicators of household wealth in the AHRI population surveillance area. For the present analysis, ‘wealthy’ households were classified as those having at least 15 assets and poor households were those with <15 assets. Household assets were included in adjusted models as a proxy for household income given the degree of missingness in the income variable.

^‡^ HIV status missing in 4

^‡‡^ HIV status missing in 9

**Table S3. Regression model outputs**

|  |  | **Basic model** | | **Adjusted model** | | **Adjusted model: sensitivity analysis**^§^ | |
| --- | --- | --- | --- | --- | --- | --- | --- |
| **Infant feeding knowledge (early postnatal interview, *n*=1693)** | | | | | | | |
|  | | **RR** | ***p*-value** | **RR** | ***p*-value** | **RR** | ***p*-value** |
|  | | **(95% CI)** |  | **(95% CI)** |  | **(95% CI)** |  |
| HIV status | |  |  |  |  |  |  |
|  | Women not living with HIV | 1 (base) |  | 1 (base) |  | 1 (base) |  |
|  | Women living with HIV | 1.09 | <0.001 | 1.08 | <0.001 | 1.08 | <0.001 |
|  |  | (1.07, 1.11) |  | (1.06, 1.09) |  | (1.06, 1.10) |  |
| Maternal age* | | 1.01 | <0.001 | 1.00 | <0.001 | 1.00 | <0.001 |
|  | | (1.00, 1.01) |  | (1.00, 1.01) |  | (1.00, 1.00) |  |
| Education | |  |  |  |  |  |  |
|  | Less than high school | 1 (base) |  | 1 (base) |  | 1 (base) |  |
|  | High school or above | 0.99 | 0.612 | 1.01 | 0.629 | 1.00 | 0.993 |
|  |  | (0.98, 1.01) |  | (0.98, 1.03) |  | (0.96, 1.04) |  |
| Exposed to CQI during pregnancy | |  |  |  |  |  |  |
|  | Unexposed |  |  | 1 (base) |  | 1 (base) |  |
|  | Exposed |  |  | 0.99 | 0.768 | 1.01 | 0.591 |
|  |  |  |  | (0.96, 1.03) |  | (0.97, 1.06) |  |
| Calendar time* | |  |  | 1.01 | 0.229 | 1.00 | 0.876 |
|  | |  |  | (1.00, 1.02) |  | (0.99, 1.01) |  |
| Employment | |  |  |  |  |  |  |
|  | Employed/ other |  |  | 1 (base) |  | 1 (base) |  |
|  | Unemployed |  |  | 0.99 | 0.339 | 1.02 | 0.254 |
|  |  |  |  | (0.97, 1.01) |  | (0.99, 1.05) |  |
| Relationship status | |  |  |  |  |  |  |
|  | Married/ living with partner/ other |  |  | 1 (base) |  | 1 (base) |  |
|  | Not married, not living with partner |  |  | 0.98 | 0.224 | 0.99 | 0.730 |
|  |  |  |  | (0.96, 1.01) |  | (0.95, 1.04) |  |
| Number of children | |  |  |  |  |  |  |
|  | More than 1 child |  |  | 1 (base) |  | 1 (base) |  |
|  | Has one child |  |  | 0.96 | 0.005 | 0.95 | 0.001 |
|  |  |  |  | (0.93, 0.99) |  | (0.93, 0.98) |  |
| Household assets | |  |  |  |  |  |  |
|  | ≥15 assets |  |  | 1 (base) |  |  |  |
|  | <15 assets |  |  | 1.04 | 0.010 |  |  |
|  |  |  |  | (1.01, 1.06) |  |  |  |
| Household income | |  |  |  |  |  |  |
|  | Family income ≥R2000 |  |  |  |  | 1 (base) |  |
|  | Family income <R2000 |  |  |  |  | 0.96 | <0.001 |
|  |  |  |  |  |  | (0.95, 0.98) |  |
| **Infant feeding practice (6-week postnatal interview, *n*=471)** | | | | | | | |
|  | | **RR** | ***p*-value** | **RRR** | ***p*-value** | **RRR** | ***p*-value** |
|  | | **(95% CI)** |  | **(95% CI)** |  | **(95% CI)** |  |
| **Exclusive breastfeeding** | | 1 (base) |  | 1 (base) |  | 1 (base) |  |
| **Mixed feeding vs exclusive breastfeeding** | |  |  |  |  |  |  |
| HIV status | |  |  |  |  |  |  |
|  | Women not living with HIV | 1 (base) |  | 1 (base) |  | 1 (base) |  |
|  | Women living with HIV | **0.22** | <0.001 | **0.22** | <0.001 | **0.26** | 0.002 |
|  |  | (0.12, 0.41) |  | (0.11, 0.43) |  | (0.11, 0.61) |  |
| Maternal age* | | 0.98 | 0.306 | 1.03 | 0.457 | 1.05 | 0.433 |
|  | | (0.95, 1.02) |  | (0.95, 1.11) |  | (0.92, 1.20) |  |
| Education | |  |  |  |  |  |  |
|  | Less than high school | 1 (base) |  | 1 (base) |  | 1 (base) |  |
|  | High school or above | 0.9 | 0.746 | 0.78 | 0.329 | 0.97 | 0.927 |
|  |  | (0.52, 1.60) |  | (0.47, 1.29) |  | (0.45, 2.05) |  |
| Infant feeding knowledge | | 0.50 | 0.008 | 0.56 | 0.058 | 0.79 | 0.530 |
|  | | (0.30, 0.84) |  | (0.31, 1.02) |  | (0.38, 1.65) |  |
| Exposed to CQI during pregnancy | |  |  |  |  |  |  |
|  | Unexposed |  |  | 1 (base) |  | 1 (base) |  |
|  | Exposed |  |  | 0.83 | 0.735 | 1.23 | 0.753 |
|  |  |  |  | (0.29, 2.38) |  | (0.34, 4.38) |  |
| Calendar time^*^ | |  |  | 0.86 | 0.080 | 0.78 | 0.037 |
|  | |  |  | (0.72, 1.02) |  | (0.62, 0.99) |  |
| Employment | |  |  |  |  |  |  |
|  | Employed/ other |  |  | 1 (base) |  | 1 (base) |  |
|  | Unemployed |  |  | 0.77 | 0.585 | 0.60 | 0.191 |
|  |  |  |  | (0.31, 1.95) |  | (0.28, 1.29) |  |
| Relationship status | |  |  |  |  |  |  |
|  | Married/ living with partner/ other |  |  | 1 (base) |  | 1 (base) |  |
|  | Not married, not living with partner |  |  | **3.07** | 0.005 | 3.25 | 0.056 |
|  |  |  |  | (1.41, 6.65) |  | (0.97, 10.93) |  |
| Number of children | |  |  |  |  |  |  |
|  | More than 1 child |  |  | 1 (base) |  | 1 (base) |  |
|  | Has one child |  |  | 2.45 | 0.090 | 4.16 | 0.027 |
|  |  |  |  | (0.87, 6.89) |  | (1.18, 14.68) |  |
| Household assets | |  |  |  |  |  |  |
|  | ≥15 assets |  |  | 1 (base) |  |  |  |
|  | <15 assets |  |  | 0.85 | 0.732 |  |  |
|  |  |  |  | (0.33, 2.20) |  |  |  |
| Household income | |  |  |  |  |  |  |
|  | Family income ≥R2000 |  |  |  |  | 1 (base) |  |
|  | Family income <R2000 |  |  |  |  | 0.67 | <0.001 |
|  |  |  |  |  |  | (0.57, 0.78) |  |
| **Not currently breastfeeding vs exclusive breastfeeding** | |  |  |  |  |  |  |
| HIV status | |  |  |  |  |  |  |
|  | Women living with HIV | 1 (base) |  | 1 (base) |  | 1 (base) |  |
|  | Women not living with HIV | **2.62** | <0.001 | **2.78** | <0.001 | **3.90** | <0.001 |
|  |  | (1.71, 4.02) |  | (1.78, 4.34) |  | (2.44, 6.23) |  |
| Maternal age* | | 0.98 | 0.211 | 0.98 | 0.427 | 0.98 | 0.440 |
|  | | (0.95, 1.01) |  | (0.94, 1.03) |  | (0.95, 1.02) |  |
| Education | |  |  |  |  |  |  |
|  | Less than high school | 1 (base) |  | 1 (base) |  | 1 (base) |  |
|  | High school or above | 1.35 | 0.105 | 1.24 | 0.245 | 1.12 | 0.510 |
|  |  | (0.94, 1.94) |  | (0.86, 1.78) |  | (0.80, 1.55) |  |
| Infant feeding knowledge | | 0.75 | 0.128 | 0.76 | 0.229 | 0.63 | 0.094 |
|  | | (0.52, 1.09) |  | (0.49, 1.19) |  | (0.37, 1.08) |  |
| Exposed to CQI during pregnancy | |  |  |  |  |  |  |
|  | Unexposed |  |  | 1 (base) |  | 1 (base) |  |
|  | Exposed |  |  | 1.00 | 0.991 | 1.13 | 0.776 |
|  |  |  |  | (0.52, 1.92) |  | (0.49, 2.62) |  |
| Calendar time* | |  |  | 0.97 | 0.665 | 0.98 | 0.805 |
|  | |  |  | (0.86, 1.10) |  | (0.84, 1.15) |  |
| Employment | |  |  |  |  |  |  |
|  | Employed/ other |  |  | 1 (base) |  | 1 (base) |  |
|  | Unemployed |  |  | **0.39** | 0.005 | **0.40** | 0.019 |
|  |  |  |  | (0.20, 0.76) |  | (0.19, 0.86) |  |
| Relationship status | |  |  |  |  |  |  |
|  | Married/ living with partner/ other |  |  | 1 (base) |  | 1 (base) |  |
|  | Not married, not living with partner |  |  | 1.08 | 0.553 | 0.98 | 0.893 |
|  |  |  |  | (0.83, 1.41) |  | (0.78, 1.24) |  |
| Number of children | |  |  |  |  |  |  |
|  | More than 1 child |  |  | 1 (base) |  | 1 (base) |  |
|  | Has one child |  |  | 1.40 | 0.116 | 1.22 | 0.406 |
|  |  |  |  | (0.92, 2.14) |  | (0.76, 1.94) |  |
| Household assets | |  |  |  |  |  |  |
|  | ≥15 assets |  |  | 1 (base) |  |  |  |
|  | <15 assets |  |  | 0.98 | 0.879 |  |  |
|  |  |  |  | (0.74, 1.29) |  |  |  |
| Household income | |  |  |  |  |  |  |
|  | Family income ≥R2000 |  |  |  |  | 1 (base) |  |
|  | Family income <R2000 |  |  |  |  | 0.65 | 0.031 |
|  |  |  |  |  |  | (0.44, 0.96) |  |

^§^ Household income was substituted for household assets.

* Calendar time was defined as time step (according to the stepped-wedge study design) at delivery and included as a continuous variable for parsimony. Maternal age at delivery was also a continuous variable in the model.

CQI, continuous quality improvement; RR, risk ratio (Poisson model); RRR, relative risk ratio (multinomial regression model)
